# Systematic Review—Role of MRI in Cervical Cancer Staging

**DOI:** 10.3390/cancers16111983

**Published:** 2024-05-23

**Authors:** Jason Chen, Yu Xuan Kitzing, Glen Lo

**Affiliations:** 1 Department of Radiology, Sir Charles Gairdner Osborne Park Health Care Group, Perth 6009, Australia; 2 Department of Radiology, Royal Prince Alfred Hospital, Sydney 2050, Australia; yuxuan.kitzing@health.nsw.gov.au

**Keywords:** cervical cancer, MRI, staging

## Abstract

**Simple Summary:**

Cervical cancer is the fourth most common cancer in women and its staging is classified by the International Federation of Gynecology and Obstetrics staging, which was updated in 2018. This is based on a combination of histopathology, clinical examination and radiographical findings. MRI plays a critical role due to its superior soft tissue resolution. The aim of our systematic review was to assess the diagnostic accuracy of MRI in the staging of cervical cancer. The literature from the last 5 years showed that MRI had a high accuracy and sensitivity in assessing stromal invasion, high accuracy and specificity in assessing pelvic sidewall involvement, and high accuracy, specificity and negative predictive value in assessing bladder and rectal involvement.

**Abstract:**

A systematic review of the diagnostic accuracy of MRI in the staging of cervical cancer was conducted based on the literature from the last 5 years. A literature search was performed in the Cochrane Library, EMBASE, MEDLINE and PubMed databases using the MeSH terms “cervical cancer”, “MRI” and “neoplasm staging”. A total of 110 studies were identified, of which 8 fit the inclusion criteria. MRI showed adequate accuracy (74–95%) and high sensitivity (92–100%) in assessing stromal invasion. The data for MRI in terms of assessing vaginal and pelvic side wall involvement were wide ranging and inconclusive. In assessing lymph node metastasis, MRI showed an adequate accuracy (73–90%), specificity (75–91%) and NPV (71–96%) but poor sensitivity (52–75%) and PPV (52–75%). MRI showed high accuracy (95%), sensitivity (78–96%), specificity (87–94%), and NPV (98–100%) but poor PPV (27–42%) in detecting bladder involvement. There was a paucity of data on the use of MRI in assessing rectal involvement in cervical cancer. Overall, the literature was heterogenous in the definitions and language used, which reduced the comparability between articles. More research is required into the diagnostic accuracy of MRI in the staging of cervical cancer and there must be increased consistency in the definitions and language used in the literature.

## 1. Introduction

Globally, cervical cancer is the fourth most common cancer in women, with around 660,000 new cases and around 350,000 deaths in 2022 [1]. Since the discovery of the relationship between human papillomavirus (HPV) and cervical cancer by Dr Harald zur Hausen in the 1980s [2], the approval of the first HPV vaccine in 2006, and ongoing screening programs, the incidence and mortality associated with cervical cancer have more than halved [3]. However, this is mainly in developed countries due to the availability of resources for such programs, whereas around 90% of cervical cancer deaths in 2015 were in low-income and middle-income countries [3].

The pre-treatment staging of cervical cancer is pivotal in determining the patient’s treatment trajectory, and MRI contributes to this planning [4,5]. Previously, the International Federation of Gynecology and Obstetrics (FIGO) 2009 staging was based solely on clinical examination and did not account for nodal status, which resulted in inaccuracies in advanced disease identified on imaging [6,7]. With the revised FIGO 2018 staging, there has been the incorporation of imaging findings into the pre-treatment staging of cervical cancer [8]. Local staging of cervical cancer depends on determining the depth of stromal invasion, size of the lesion, invasion into parametrium, vagina or pelvic sidewall, bladder/rectal involvement and lymph node metastasis (LNM) [9].

**Figure 1:**
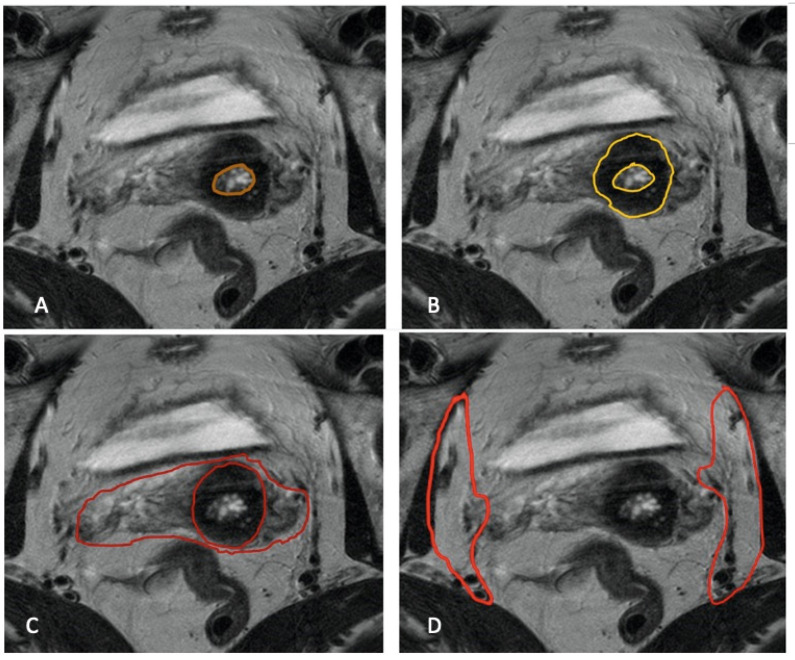
Normal MRI cervical appearances with relevant structures demarcated for illustration purpose—(**A**) cervical mucosa (circled by orange) is T2 hyperintense; (**B**) normal cervical stroma (circled by yellow) is T2 dark and regular; (**C**) parametrium with fatty tissue (circled by red) is T2 intermediate (gray) in signal due to the rich vascular plexus; and (**D**) pelvic sidewall (circled by vermillion) is adjacent to and inclusive of the obturator internus muscles.

**Figure 2:**
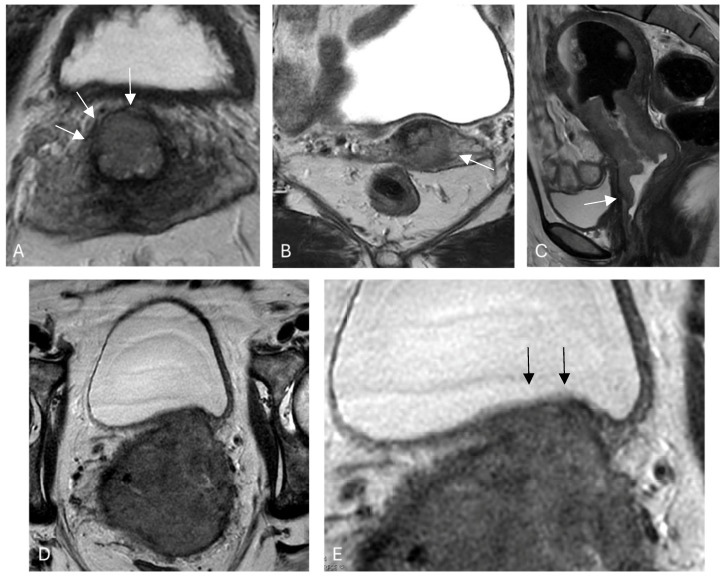
Typical MRI appearances of cervical cancer with correlation to the FIGO stages—(**A**) intact but thinned T2 dark cervical stromal ring (arrows); (**B**) parastromal invasion by the cancer with loss of the dark T2 ring focally and extension of the tumor into the parametrium; (**C**) vaginal involvement (arrow); (**D**) urinary bladder detrusor invasion but with preservation of the bladder mucosa (not T4); and (**E**) closeup of intact bladder mucosa (arrows).

Pelvic MRI is the imaging modality of choice for cervical cancer’s local staging due to its superior soft tissue contrast, relatively high spatial resolution, and the lack of ionizing radiation [10]. MRI has also shown high reproducibility between observers and high accuracy when compared with histopathology samples [11,12]. The normal MRI appearance of the cervix (Figure 1) includes a T2 dark (black) stromal ring. When cancer is present and locally invasive (Figure 2), it can disrupt the stromal ring and extend into the parametrium.

The majority of cervical cancer cases are low-stage tumors such as FIGO stage IB and II. MRI allows for identification of cervical cancer cases that are at higher risk of a positive surgical margin (Ib3, IIa2, IIb). The higher-risk group may be offered upfront chemoradiotherapy to avoid the morbidity associated with postoperative adjuvant chemoradiation, whereas lower-stage cases can be confidently offered primary surgery, including fertility preservation options in the appropriate patient group [13].

### 1.1. Histological Subtypes

The most common histological subtypes of cervical cancer are squamous cell carcinoma (SCC) and adenocarcinoma (AC), which account for around 70% and 25% of all cervical cancers, respectively [14]. The remaining 5% of cervical cancers include subtypes such as adenosarcomas, carcinosarcomas, clear cell carcinoma, germ cell tumors and neuroendocrine tumors.

The majority of cervical SCC cases are associated with human papillomavirus (HPV), especially genotypes 16 and 18—the major targets for vaccination in the prevention of HPV-related cervical cancer [13]. However, HPV vaccination and screening have a less pronounced effect on cervical AC, with 15 to 20% of AC cases being HPV negative and the proportion of cervical cancers that are AC now increasing from 5% to 8–27% [15].

### 1.2. Clinical Management—Surgery, Radiation and Chemotherapy

Cervical cancer treatment options include surgery, radiation and chemotherapy, and management decisions are mainly based on staging, which can be via the FIGO 2018 or TNM classifications. The FIGO 2018 staging is more commonly used [3,8,16]. The FIGO 2018 staging determinants include the depth of stromal invasion, tumor size, parametrial spread, vaginal and pelvic side wall involvement, associated renal complications, lymph node spread, bladder and/or rectal involvement and solid organ metastasis.

Surgery is the mainstay management in the early stages of cervical cancer. Surgical techniques with curative intent include simple or radical hysterectomy with pelvic lymphadenectomy [8,9]. Fertility-sparing surgery with trachelectomy may be offered with appropriate patient selection [9]. However, for tumors greater than 4 cm or with parametrial invasion (FIGO IIB or higher), the patient will proceed with chemoradiation to avoid the morbidity associated with postoperative adjuvant radiation [9]. In selected patients with advanced-stage IVA disease, pelvic exenteration may be suitable [8].

In our institution, a postoperative Gynecologic Oncology Group (GOG) score [17] is used in conjunction with the FIGO 2018 staging to determine the most suitable treatment regime. The GOG score is calculated by incorporating the postoperative histopathological findings of the clinical tumor size, capillary/lymphatic space involvement and depth of tumor invasion [17]. These factors have been shown to be independent prognostic factors for a 3-year disease free interval in patients with cervical cancer [17]. This has been used by many institutions, and it has been shown that patients with a GOG score <70 are associated with good recurrence-free survival without adjuvant therapy [18]. However, patients with a GOG score >120 have a 41% risk of recurrence without adjuvant therapy after radical hysterectomy and the standard of care for patients with a GOG score between 70 and 120 is less clear [18,19].

### 1.3. Decision for Upfront Radiation Therapy

A limitation of the GOG score is that it is a postoperative score that guides the use of adjuvant radiotherapy. Preoperative clinical examination and MRI can be used to identify patients expected to have a high GOG score based on their estimated tumor size and depth of invasion and to select the patients for upfront chemoradiation instead of surgery [20].

As such, the diagnostic accuracy of MRI plays a critical role in directing treatment for patients with local staging of cervical cancer. We aimed to systematically review the diagnostic accuracy of MRI in the staging of cervical cancer, with a specific focus on FIGO IIb (with parametrial involvement but not to pelvic sidewall) diagnostic performance, as it may preoperatively influence the estimated GOG score and redirect patients from surgery to radiation therapy.

## 2. Materials and Methods

### 2.1. Literature Search

**Figure 3:**
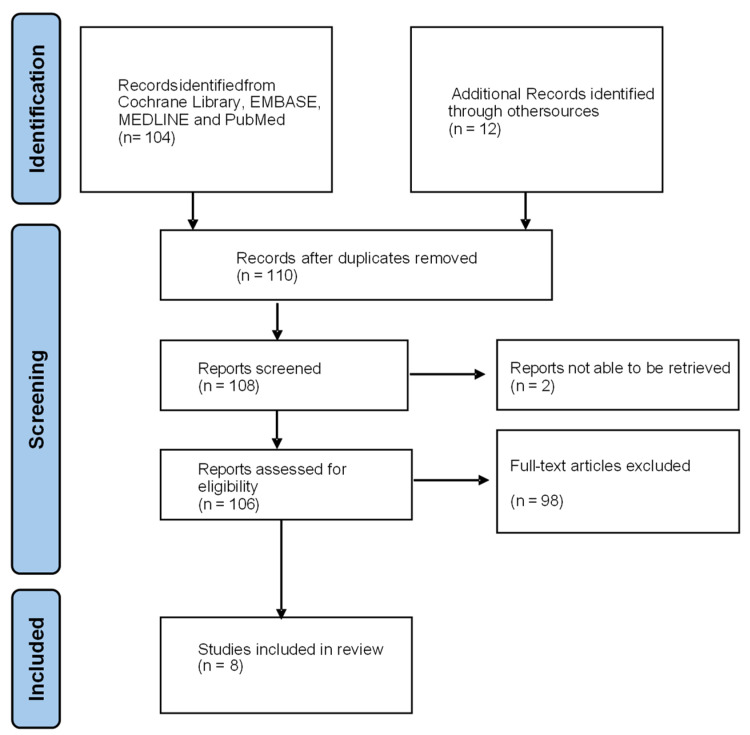
Flowchart outlining the study selection process.

A literature search was performed in the Cochrane Library, EMBASE, MEDLINE and PubMed for all the available English full-text articles on human subjects from the 1 January 2018 to the 31 December 2023. The terms used as medical subject headings (MeSH) included “cervical cancer”, “MRI”, and “neoplasm staging”. These keywords were identified through several searches, which will encapsulate the required studies. The abstracts were screened for eligibility by the first author. Additional eligible studies from the citations of the original research articles and review articles were also added (Figure 3). A gynecological imaging specialist was appointed to review any articles where there was a disagreement.

This study was conducted by following the Preferred Reporting Items for Systematic Reviews and Meta-Analyses (PRISMA) and in accordance with the Declaration of Helsinki. The study protocol was not registered. No new data were created or analyzed in this study. Data sharing is not applicable to this article.

### 2.2. Inclusion Criteria

1. Only original articles with reported data were included.
2. The studies were limited to adult females with a histopathological diagnosis of cervical cancer.
3. Preoperative MRI imaging compared to preoperative clinical assessment or postoperative histopathological diagnosis.
4. Retrospective and prospective studies were included.

### 2.3. Exclusion Criteria

1. Reviews and conference articles were excluded.
2. Studies of patients who have had preoperative radiotherapy and chemotherapy after their initial MRI were excluded.
3. Studies that focused on recurrent cervical cancer or cervical cancer post treatment follow up were excluded.
4. Studies on MRI radiomics were excluded.

### 2.4. Data Evaluation

The obtained data included (1) the year of publication, (2) reference standard, (3) accuracy, (4) sensitivity, (5) specificity, (6) positive predictive value (PPV), and (7) negative predictive value (NPV), as shown in Table 1 and Table 2. In some papers, the accuracy, sensitivity, specificity, PPV and/or NPV were not reported but there were sufficient data reported for these measures to be estimated. However, in a few of the studies, there was insufficient data to allow for this, so the missing parameters were left empty.

## 3. Results

Table 1 shows the estimated diagnostic accuracy, sensitivity, specificity, PPV and NPV of MRI in determining the stromal and parametrial invasion of cervical cancer, which correlates up to the FIGO 2018 stage IIB. Table 2 shows the same parameters but in determining pelvic side wall invasion, vaginal involvement, lymph node metastases and bladder and rectal involvement, which would correlate with higher-stage cervical cancer from FIGO 2018.

Whilst the majority of studies conformed to the aforementioned parameters, which are in line with the FIGO 2018 staging terminology, Ran et al. instead used para-uterine infiltration as a parameter [22]. This was defined as all the para-uterine soft tissue involvement, para-uterine lymph node metastases and tumor emboli into the para-uterine vessels, which made their data incomparable with the other studies.

Steiner et al. utilized a combination reference standard of histopathology in patients who underwent surgery as well as a consensus reference standard [6]. This was created in a joint meeting by two radiologists and two gynecologists based on all the available information of the patient from clinical examination and imaging studies. This was retrospective in nature, with at least a year between the PET/MRI analysis and consensus evaluation, with changes in size and/or SUVmax seen as malignancy.

The studies varied in the experience of the person reporting the MRI. Knoth et al. [7] reported MRI interpreted by either radiologists or radiation oncologists experienced in the treatment of gynecologic malignancies. Steiner et al. [6] used a senior abdominal radiologist with more than 10 years of experience in MRI and 2 years of experience in PET fusion imaging. A review of MRI alone was performed by another senior radiologist with more than 10 years’ experience in abdominal MRI. Ran et al.’s [22] MRI findings were evaluated by a radiologist with 7 years’ experience in pelvic MRI diagnosis and a senior radiologist with 20 years’ experience in pelvic MRI diagnosis. For Matsumoto et al. [23], all the MR examinations were interpreted independently by 2 board-certified radiologists with 24 years and 11 years of experience in gynecologic cancer imaging. For Rockall et al. [25], all the imaging scans were read by one local and two central radiologists. All the radiologists were accredited core members of the gynae-oncology multi-disciplinary team and/or PET/CT experts. Smits et al. [21] reported MRI assessed by experienced radiologists, with the type not specified. Anfinan [24] and Zhu et al. [26] did not specify the experience of their radiologist.

### 3.1. Stromal Invasion

Three articles reported on stromal invasion; however, their primary outcomes were heterogenous, which reduced the comparability and we were unable to pool (Table 1). Smit et al. investigated the performance of MRI in early disease staging (FIGO 2009 stage IA1, IA2, IB1 and IIA1), reporting 94.8% sensitivity, 38.5% specificity, 94.8% PPV and 38.5% NPV [21]. Ran et al. investigated the ability of MRI in detecting FIGO 2018 stage IB and IIA, which showed an accuracy, sensitivity and specificity of 88.6%, 98.8% and 97.0% for stage IB and 94.8%, 100% and 95.5% for stage IIA [22]. Steiner et al. was the only study to use only the TNM staging and looked at MRI in detecting deep stromal invasion (defined as ≥50% of cervical stromal thickness), which showed a 95% accuracy, 100% sensitivity, 91% specificity, 90% PPV and 100% NPV [6].

### 3.2. Parametrial Invasion

Five articles reported on parametrial invasion (Table 1) [6,21,22,23,24]. Four of these articles used parametrial invasion as a measurable outcome, and for the reference standard, three of these articles used histopathology and one used clinical examination. The remaining article by Ran et al. looked at the performance of MRI in diagnosing FIGO 2018 IIB cervical cancer and assessing para-uterine involvement, with histopathology as the reference standard.

Smits et al. reported a sensitivity, specificity, PPV and NPV of 33.3%, 96.3%, 25% and 97.5%, respectively, for MRI in detecting parametrial involvement when compared to histopathology [21]. This was the lowest reported sensitivity but the highest specificity for detecting parametrial invasion. The accuracy was not reported and there was insufficient data to allow for its calculation. Matsumoto et al. investigated the ability of two readers in detecting parametrial invasion on T2WI and DWI MRI sequences when used independently and in conjunction with each other [23]. The use of T2WI and DWI together in detecting parametrial invasion produced the best results, with a 66.7–75% sensitivity, 93.7–95.1% specificity, 89.5–89.9% accuracy, 75–76.9% PPV and 92.1–93.7% NPV when compared to histopathology. Steiner et al. reported the 100% sensitivity, 46% specificity, 63% accuracy, 46% PPV and 100% NPV of MRI in detecting parametrial invasion when compared to histopathology [6].

Anfinan was the only study that compared MRI findings with clinical examination and reported a 72% sensitivity, 82% specificity, 96% PPV and 33% NPV [24]. Sufficient data were available to calculate an overall accuracy of 74% for MRI in detecting parametrial invasion when referenced to clinical examination.

Ran et al. reported on the accuracy of MRI in detecting FIGO 2018 stage IIB cervical cancers (which have parametrial involvement but not up to the pelvic wall), with an accuracy of 92.7%, sensitivity of 85.7% and specificity of 92.5% [22]. This is comparable to the previously mentioned articles; however, Ran et al. also reported on MRI in detecting para-uterine infiltration, with a 56.9% sensitivity, 74.1% specificity, 90.7% accuracy, 43.5% PPV and 92.2 NPV. However, as mentioned earlier, para-uterine infiltration was not used in any other articles and would include all the cervical cancers from FIGO 2018 stage IIB up to at least stage IIIC, which made it incomparable with other studies.

### 3.3. Vaginal Involvement

Four studies reported on vaginal involvement, with two using histopathology and two using clinical examination as a reference standard (Table 2) [6,7,22,24].

Once again, the outcome of para-uterine involvement used by Ran et al., with histopathology as the reference standard, encompasses vaginal invasion but is not directly comparable to the other studies. However, Ran et al. also looked at FIGO stage IIA detection, which is defined as cervical cancers that are limited to the upper two-thirds of the vagina without parametrial involvement, and reported a accuracy of 94.8%, sensitivity 100% and specificity 95.5% [22]. Steiner et al. was the other study that used histopathology as the reference standard and reported an accuracy of 67%, sensitivity 100%, specificity 48%, PPV 52% and NPV 100% [6].

Knoth et al. used clinical examination of vaginal wall involvement as the reference standard and reported MRI to have a sensitivity of 84.6%, specificity 90.4%, PPV 87.7% and NPV 87.9%, which resulted in a calculated accuracy of 87.8% [7]. Anfinan also used clinical examination as the reference standard and found MRI to have a sensitivity of 67%, specificity 60%, PPV 47%, NPV 77% and a calculated accuracy of 62% in detecting vaginal involvement [24].

### 3.4. Pelvic Side Wall

Only Anfinan reported on pelvic side wall invasion as a discrete parameter, with clinical examination as the reference standard (Table 2) [24]. The reported accuracy was 83.6%, sensitivity 56%, specificity 94%, PPV 77% and NPV 85%. Ran et al. did not directly report on pelvic side wall involvement but on FIGO 2018 stage IIIB, which is defined as pelvic wall invasion and/or hydronephrosis or non-functioning kidney from the tumor [22]. Ran et al. reported a 91.4% accuracy, 100% sensitivity and 91.5% specificity. However, given that Ran et al. only had two FIGO IIIB cases and by definition these included patients with renal involvement, the two studies were not directly comparable.

### 3.5. Lymph Node Metastasis

Four articles reported on the estimated diagnostic ability of MRI in detecting LNM (Table 2) and all four used histopathology as the reference standard [6,22,25]. Ran et al. reported that MRI had an estimated accuracy of 89.5%, sensitivity 55.2%, specificity 91.6%, PPV 51.6% and NPV 96.4% in detecting LNM [22]. Steiner et al. reported an accuracy of 73%, sensitivity 71%, specificity 75%, PPV 75% and NPV 71% in determining nodal staging as a part of the TNM staging system [6]. Rockall et al. reported on the use of diffusion-weighted MRI specifically in the nodal staging of patients with cervical cancer, with a sensitivity of 20%, false positive rate 3.3%, PPV 66.7% and NPV 98.4% and a calculated specificity of 96.7% and accuracy of 77.5% [25]. Zhu et al. reported on the diagnostic accuracy of CT, MRI, PET/CT and PET/MRI in detecting LNM and showed that MRI had an accuracy of 79%, sensitivity 76% and specificity 80% [26]. Although this was inferior to PET/CT, the study showed that PET/MRI had the greatest accuracy, sensitivity and specificity in detecting LNM.

### 3.6. Bladder and Rectal Involvement

Three articles reported on bladder and rectal involvement, with two using clinical examination as the reference standard and one using histopathology (Table 2) [6,7,24]. Overall, the prevalence of bladder involvement was low, with the largest study being performed by Knoth et al. [7], with only 26 patients having bladder involvement out of 538 patients who underwent cystoscopy. The prevalence of rectal invasion was even lower and the studies were not sufficiently powered to draw any meaningful conclusion regarding the diagnostic performance of MRI in detecting rectal invasion.

Knoth et al. used cystoscopy as the reference standard for bladder involvement and reported an estimated diagnostic performance of MRI in detecting bladder wall involvement, with an accuracy of 93.6%, sensitivity 96.2%, specificity 93.5%, PPV 42.4% and NPV of 99.8% [7]. The study attempted to assess rectal involvement as a separate entity, but this was underpowered and no figures were reported, as only one patient had rectal wall infiltration on rectoscopy and this was also seen on MRI.

Anfinan reported bladder and rectal involvement as separate entities, with clinical examination with cystoscopy and sigmoidoscopy being used as the respective reference standards [24]. Anfinan reported a sensitivity of 78%, specificity 87%, PPV 27% and NPV 98% for MRI in detecting cystoscopy-proven bladder involvement and a sensitivity of 75%, specificity 91%, PPV 15% and NPV 99% in detecting sigmoidoscopy-proven rectal involvement. The prevalence of rectal and bladder involvement was low, with 9 out of 150 patients who underwent cystoscopy being found to have bladder involvement and only 1 out of 149 patients who underwent sigmoidoscopy found to have rectal involvement. However, none of the patients with negative rectal involvement on MRI were found to have rectal involvement by sigmoidoscopy.

Steiner et al. was the only study to use histopathology as a reference standard for bladder and rectal involvement but reported the 2 as 1 entity as only 3 patients had bladder and/or rectal involvement [6]. Steiner et al. reported an accuracy of 97% for MRI in detecting bladder and/or rectal involvement, with all the true positives being detected by MRI but an additional false positive for rectal involvement detected.

## 4. Discussion

The increasing role of MRI in the local staging of cervical cancer and use as an adjunct in determining treatment trajectory has meant that it is important to determine its diagnostic accuracy. Our review initially aimed at looking at the diagnostic accuracy of MRI in determining the tumor depth and size, which can be used preoperatively to predict a high GOG score that would direct treatment toward chemoradiation therapy as this has been shown to have improved outcomes compared to upfront surgery with subsequent adjuvant chemoradiation therapy [27]. Of the publications from the last 5 years, only 2 articles directly investigated the diagnostic accuracy of MRI in detecting early-stage disease [21] and deep stromal invasion (Table 2) [6]. A third article reported on the diagnostic ability of MRI in detecting FIGO 2018 Stage IB and IIA cervical cancer rather than measuring stromal invasion itself [22]. The reported accuracy ranged from 74–95%, sensitivity 92–100%, specificity 39–97%, PPV 67–95%, and NPV 39–85%. Whilst the reported outcomes of these studies were heterogenous in nature and incomparable, they showed adequate diagnostic accuracy and high sensitivity overall.

Vaginal involvement limited to the upper two-thirds of the vagina defines FIGO 2018 stage II, with the absences and presence of parametrial involvement distinguishing stage IIA from IIB. There were three articles that reported directly on vaginal involvement and were comparable, with a reported accuracy of 62–88%, sensitivity 67–100%, specificity 48–90%, PPV 47–88%, and NPV 77–100% [6,7,24]. However, the studies did not specify if the vaginal involvement was confined to the upper two-thirds of the vagina or involved the lower third of the vagina and the reported results were of a wide range. In terms of parametrial invasion, four articles reported directly on parametrial invasion, with three using histopathology as the reference standard and one using clinical examination instead. Overall, these were comparable but showed a wide range, with a reported accuracy of 63–93%, sensitivity 33–100%, specificity 46–96%, PPV 25–96%, and NPV 33–98% [6,21,23,24]. Ran et al. reported on para-uterine involvement, which included para-uterine soft tissue involvement, para-uterine LNM and tumor emboli into para-uterine vessels, and the wide encompassing nature of this meant that it was not directly comparable to any other article [22]. Whilst Ran et al. also reported on the diagnostic accuracy of MRI in detecting various FIGO 2018 stages (Table 1 and Table 2), these were once again not comparable to other studies.

Anfinan was the only study that directly reported on pelvic side wall involvement, with clinical examination as the reference standard and an accuracy of 84%, sensitivity 56%, specificity 94%, PPV 77% and NPV 85% reported [24]. Whilst Ran et al. reported the accuracy of MRI in detecting FIGO stage IIIB, with an accuracy of 91%, sensitivity 100% and specificity 97%, the inclusion of hydronephrosis or nonfunctioning kidney in additional to pelvic side wall involvement reduced the comparability [22,24].

Four studies reported on LNM, with histopathology as the reference standard [6,22,25,26]. These were comparable and reported an accuracy range of 73–90%, sensitivity 20–76%, specificity 75–91%, PPV 52–75%, and NPV 71–96%. These suggested an adequate accuracy, specificity and NPV but a poor sensitivity and PPV. In clinical practice, LNM is better determined by PET/CT; however, there is an emerging role of PET/MRI, which has been shown to have superior sensitivity, specificity and accuracy, even when compared to PET/CT [26].

There were also three studies that reported on bladder and rectal involvement, with two using clinical examination via cystoscopy or rectoscopy as the reference standard [7,24] and one using histopathology [6]. In terms of detecting bladder invasion, MRI, when compared to clinical examination, reported an accuracy of 95%, sensitivity 78–96%, specificity 87–94%, PPV 27–42% and NPV 98–100%. These results indicated that MRI had a high accuracy, sensitivity, specificity and NPV in determining bladder invasion but a poor PPV. Only Anfinan reported on MRI detection of rectal invasion compared to clinical examination, with a sensitivity 75%, specificity 91%, PPV 15% and NPV 99% [24]. Steiner et al. reported on bladder and rectal invasion together and so was incomparable with other studies [6]. Whilst there were only a small number of cases, with Steiner et al. reporting only 3 patients with bladder and rectal invasion, Anfinan having 9 bladder and 1 rectal invasion and Knoth et al. with 26 cases of bladder invasion and 1 case of rectal invasion, the studies showed the high overall specificity and NPV of MRI in assessing bladder and rectal invasion.

## 5. Conclusions

Overall, the literature is heterogenous in nature, with a wide range of reported accuracy, sensitivity, specificity, PPV and NPV for MRI in determining stromal invasion, parametrial invasion, vaginal involvement, pelvic sidewall and/or associated renal complications, lymph node metastases and bladder and rectal involvement. There must be increased consistency in the language and definitions used in further research into the diagnostic accuracy of MRI in the staging of cervical cancer.

## Figures and Tables

**Table 1:** Articles that assessed the accuracy, sensitivity, specificity, positive predictive value (PPV), negative predictive value (NPV) of MRI in the assessment of stromal invasion and parametrial and pelvic wall involvement.

Study	Reference	Year	No. of Patients	Reference Standard	Accuracy (%)	Sensitivity (%)	Specificity (%)	PPV (%)	NPV (%)	Outcome
**Stromal Invasion**										
Smits et al.	[21]	2023	358	H	-	95	39	95	39	Early-stage disease
Ran et al.	[22]	2021	200	H	89	99	97	-	-	FIGO 2018 IB
Ran et al.	[22]	2021	200	H	95	100	96	-	-	FIGO 2018 IIA
Steiner et al.	[6]	2021	20	H	74	89	60	67	86	Deep stromal invasion
**Parametrial Invasion**										
Smits et al.	[21]	2023	167	H	-	33	96	25	98	Parametrial
Ran et al.	[22]	2021	200	H	93	86	93	-	-	FIGO 2018 IIB
Ran et al.	[22]	2021	200	H	91	56.9	74.1	43.5	92	Para-uterine *
Matsumoto et al.	[23]	2021	51	H	90	67–75	94–95	75–77	92–94	Parametrial
Steiner et al.	[6]	2021	33	H	63	100	46	46	100	Parametrial
Anfinan	[24]	2019	152	CE	74	72	82	96	33	Parametrial

* Para-uterine is defined as including para-uterine soft tissue involvement, para-uterine LNM and tumor emboli into para-uterine vessels; H: histopathological; CE: clinical examination.

**Table 2:** Articles that assessed the accuracy, sensitivity, specificity, positive predictive value (PPV) and negative predictive value (NPV) of MRI in the assessment of pelvic side wall involvement, vaginal involvement, lymph node metastases and bladder and rectal involvement.

Study	Reference	Year	No. of Patients	Reference Standard	Accuracy	Sensitivity	Specificity	PPV	NPV	Outcome
**Vaginal Involvement**										
Ran et al.	[22]	2021	200	H	91	57	74	44	92	Para-uterine *
Ran et al.	[22]	2021	200	H	95	100	96	-	-	FIGO Stage IIA
Steiner et al.	[6]	2021	33	H	67	100	48	52	100	VI
Knoth et al.	[7]	2020	1338	CE	88	85	90	88	88	VI
Anfinan	[24]	2019	145	CE	62	67	60	47	77	VI
**Pelvic Side Wall and/or Associated Renal Complications**										
Ran et al.	[22]	2021	200	H	91	100	97	-	-	FIGO stage IIIB
Anfinan	[24]	2019	152	CE	84	56	94	77	85	Pelvic side wall
**Lymph Node Metastases**										
Ran et al.	[22]	2021	200	H	90	55	92	52	96	LNM
Rockall et al.	[25]	2021	40	H	78	20	97	67	78	DW MRI
Steiner et al.	[6]	2021	33	H	73	71	75	75	71	LNM
Zhu et al.	[26]	2021	196	H	79	76	80	-	-	LNM
**Bladder and Rectal Involvement**										
Steiner et al.	[6]	2021	4	H	97	-	-	-	-	BI and RI
Knoth et al.	[7]	2020	548	CE	94	96	94	42	100	BI
Anfinan	[24]	2019	152	CE	-	78	87	27	98	BI
Anfinan	[24]	2019	152	CE	-	75	91	15	99	RI

* Para-uterine is defined as including para-uterine soft tissue involvement, para-uterine LNM and tumor emboli into para-uterine vessels; H: histopathological; CE: clinical examination; VI: vaginal involvement; LNM: lymph node metastases; BI: bladder involvement; RI: rectal involvement.

## Data Availability

No new data were created or analyzed in this study. Data sharing is not applicable to this article.
